# Many Internal Improvements

**Published:** 1893-10-28

**Authors:** 


### MIDDLESEX HOSPITAL.

### MANY INTERNAL IMPROVEMENTS.

The Middlesex Hospital was reopened a few weeks back after having been entirely closed for three months, during which time important improvements have been carried out. Fifteen wards have been refloored with teak; of the remaining wards three were treated in a similar way last year, and four several years back; there are, therefore, now no wards without teak floors. When the old boards were removed, and before the new teak boards were laid down, the spaces between the floor timbers were carefully cleaned out and joints washed with a solution of perchloride of mercury (1 in 1,000). The surface of the teak boards, which are laid in four-inch widths and secret nailed, was wax polished.

In all the wards new fireplaces, specially designed, have been put to replace the old wasteful grates, the greater part of the heat from which passed up into the chimneys. These grates are a development of that designed by Mr. Pridgin Teale (author of "Dangers to Health"), and are what is known as the front hob pattern. The grate consists of fire brick back and sides, and a bottom grid on which the fire is made, and below which is an ash-pan. There are no front bars at all; a raised hearth or hob projects in front of the fire, and has a space underneath through which the ash-pan is drawn out. The mantelpieces are formed of small enamelled bricks with a teak shelf. At the side of each grate is a recess formed of the same enamelled bricks, in which is a gas stove and a stand for a kettle. The fumes from the gas stove pass off into the smoke flue. The burner, which is an atmospheric one, has a small pilot light, which is kept constantly burning, and by means of a spring the weight of a kettle placed on the stove presses the top down and lights the burners. When the kettle is removed the top springs back again, and all the burners, except the pilot light, are extinguished.

The ward sculleries have throughout been floored with teak in the same manner and with the same antiseptic precautions as the wards. The old baths have been removed, and the sculleries fitted with new teak dressers and lockers for the nurses, porcelain sinks, lead sinks for portable baths, and new cooking stoves. To take the place of the old baths in the sculleries, a set of four new bath-rooms were last year formed in the basement, lined with glazed tiles, and fitted with porcelain baths.

The opportunity afforded by the closing of the hospital, in order to carry out the works described above, was taken to repaint and cleanse the whole of the interior, to paint the exterior, and to complete the alterations to the drains begun last year. The old drains have been almost entirely removed and relaid either with tested stoneware pipes or, where drains pass under any part of the building, with heavy cast-iron pipes, and all were carefully tested with water before being covered in.

Plans have been prepared for a new operation theatre, with anæsthetic room, surgeon's room, and recovery room adjoining; also a small operation room for special operations. This work will be begun early next year.

